# Days alive and out of hospital after burr-hole drainage for chronic subdural haematoma: a national cohort study using Hospital Episode Statistics in England

**DOI:** 10.1136/bmjopen-2025-114095

**Published:** 2026-04-13

**Authors:** Daniel Thompson, Adam Wahba, Benjamin Davies, Adam Williams, Peter John Hutchinson, Daniel Stubbs, Adel Helmy, David A Cromwell

**Affiliations:** 1 Department of Clinical Neurosciences, University of Cambridge, Cambridge, UK; 2 Department of Neurosurgery, NHS North Bristol NHS Trust, Bristol, UK; 3 Department of Neurosurgery, Royal Hallamshire Hospital, Sheffield, UK; 4 Perioperative, Acute, Critical, and Emergency Care Section, Department of Medicine, University of Cambridge, Cambridge, UK; 5 Department of Health Services Research and Policy, London School of Hygiene & Tropical Medicine, London, UK

**Keywords:** Neurosurgery, Risk Factors, Longitudinal studies, Health Equity, Health policy

## Abstract

**Abstract:**

**Objectives:**

The objective of this study was to investigate the utility of the days alive and out of hospital (DAOH) metric within a cohort of patients undergoing burr-hole drainage of a chronic subdural haematoma (CSDH). We evaluate the validity of the DAOH metric in a national CSDH cohort and examine how the DAOH metric compares to its constituent outcomes (mortality and hospital bed days) at an organisational level.

**Methods:**

Retrospective cohort study using Hospital Episode Statistics data linked to the national death registry to identify patients who underwent burr-hole drainage of CSDH in English National Health Service neurosurgical units between 1 April 2013 and 31 March 2020. Construct validity was assessed by measuring the patterns of DAOH across categories of known perioperative risk factors. Variation between units in the risk-adjusted values for DAOH, postoperative mortality and days in hospital was explored using funnel plots. Linear regression and logistic regression were used to derive the risk-adjusted rates.

**Results:**

Overall, 16 450 patients who underwent at least one burr-hole drainage of CSDH were identified during the time period. The median 30-day DAOH was 16 (IQR, 0–24); the median for the 90-day DAOH was 74 (42–84), and was better at measuring the complete stay associated with the index admission. Worse 90-day DAOH values were associated with older age, increasing comorbidities and greater frailty. Risk-adjusted 90-day DAOH values for neurosurgical units varied more markedly than for its constituent outcomes.

**Conclusions:**

The 90-day DAOH looks to be a valid outcome metric for patients undergoing burr-hole drainage for CSDH that is feasible to derive using national hospital data. Future work should explore how to estimate a minimally important clinical difference for DAOH and evaluate its utility as an outcome measure.

**STRENGTHS AND LIMITATIONS OF THIS STUDY**

- National linked hospital administrative dataset enabled complete capture of admissions and mortality for chronic subdural haematoma (CSDH) surgery in England.
- Patient-level linkage allowed days alive and out of hospital (DAOH) to incorporate transfers and readmissions across hospitals.
- Comorbidity and frailty were used in risk adjustment with validated administrative data indices (van Walraven Elixhauser; Secondary Care Administrative Records Frailty (SCARF)).
- Administrative data, while providing large sample sizes, lack information on clinical severity/acuity measures (eg, Glasgow Coma Score (GCS)/neurological status, midline shift/haematoma features), limiting the ability to adjust for confounding.
- As a composite indicator, the interpretation of the DAOH is not straightforward and its bimodal distribution complicates the reporting of variation between patients.

## Introduction

 Days alive and out of hospital (DAOH) is a composite outcome measure that can synthesise different aspects of the patient recovery journey in a way that traditional standalone outcome metrics may not capture.^1^ It is a continuous outcome that combines mortality and length of stay including readmissions into a single figure and provides a directionally consistent metric that can help to better describe the patient’s recovery journey following an operation. It also has the advantage of having greater statistical power to detect differences between groups than binary outcomes such as postoperative mortality. Multiple studies have examined the outcome in various surgical cohorts and reported that, in these populations, it can discriminate between patients with different perioperative risk factors, sicker patients tend to have lower DAOH values, and a DAOH measured for a short timeframe (such as 30 or 90 days) is associated with outcomes over a longer timeframe, such as 1-year survival.^2^,^4^ It is also seen as a patient-centred metric because patients value time at home and avoiding hospitalisation.

The performance of DAOH has yet to be evaluated within a cranial neurosurgical population. Patients undergoing burr-hole drainage of a chronic subdural haematoma (CSDH) represent a useful test case because these patients constitute an elderly, comorbid population that can often spend many days in hospital (across different institutions).^5^,^7^ Studies have shown the relevance of DAOH to patients who have a hip fracture, and this cohort shares similarities with the population of individuals with a CSDH.^8^ CSDH is a common neurosurgical pathology defined by the presence of liquefied blood between the dura mater and arachnoid layers of the brain. Surgical management consists of evacuating the blood in order to relieve pressure on the brain. A landmark clinical trial demonstrated improvements in recurrence rates in patients who had a closed drainage system after burr-hole evacuation compared with no drain.^9^ A subsequent British multicentre, prospective cohort study extended this result by identifying a number of modifiable prognostic factors.^10^ However, there remains some uncertainty regarding the longer-term outcomes for this cohort of patients.^11^,^14^ Studies on CSDH patients have often focused on rates of postoperative mortality, recurrence and reoperation. These measures overlook other consequences such as prolonged hospitalisation and repeated readmissions, both of which patients prefer to avoid. The DAOH metric provides a way to capture these events.

The aim of this study was to investigate the validity of the DAOH outcome within a cohort of CSDH patients who had burr-hole surgery using a national administrative dataset. The dataset allows patient admissions to any National Health Service (NHS) acute hospital to be identified and provides a robust way to calculate time in hospital over defined time intervals. Specifically, we describe the distribution of DAOH at 30, 90, 180 and 365 days, and assess its construct validity by determining whether DAOH discriminates between groups of known risk factors. We examine how the DAOH metric compares to the separate constituent outcomes (mortality and hospital bed days) at an organisational level. In England, surgery for CSDH is performed in dedicated neurosurgery units, and their performance is monitored by the National Neurosurgical Audit Programme (NNAP) using national administrative hospital data.^15^ It has been suggested that the DAOH could add to traditional benchmarking metrics derived from administrative hospital data,^16^ but there is limited evidence about the potential value of the DAOH as a quality indicator.

## Methods

### Study design

This was a retrospective, observational cohort study design. The study used an extract of data from the Hospital Episode Statistics (HES) Admitted Patient Care dataset, linked to the Office for National Statistics (ONS) death register. HES is the national administrative dataset used by English hospitals in the NHS to record patient-level information about admissions and day cases.^17^ HES contains a unique identifier that allows inpatient/day case episodes related to the same person to be identified at any hospital. As well as tracking discrete admissions, it allows the definition of continuous inpatient spells that capture when patients are transferred from neurosurgical units to a local hospital prior to returning home or non-acute care. A HES record can capture up to 20 conditions (coded using the International Classification of Diseases, 10th revision (ICD-10) diagnosis codes) and up to 24 procedures (coded using the UK Office of Population Censuses and Surveys (OPCS, V.4) classification). The description of this retrospective cohort study follows the Strengthening the REporting of studies Conducted using Observational Routinely-collected health Data (RECORD), and a RECORD checklist is included in the online supplemental material.

### Data sources and cohorts

The study identified the HES records of patients admitted to 1 of the 24 NHS neurosurgical units in England between 1 April 2013 and 31 March 2020 (7 years), with the cohort being limited to patients classified as a neurosurgical admission (specialty code 150). The OPCS-4 procedure code ‘A411’ was used to identify patients who had undergone the ‘evacuation of subdural haematoma’ in combination with OPCS-4 code ‘Y47’, which specifies a burr-hole approach to the procedure.

### Calculation of days alive and out of hospital

The study used the DAOH definition described by Myles *et al*^2^ and was defined for four different follow-up periods (30, 90, 180, 365 days). To obtain the number of days not spent in hospital, the initial postoperative length of stay and any subsequent hospital stays within the defined period were subtracted from its overall duration. The outcome did not include any days spent in a non-hospital care facility. If a person died during the specified period, the DAOH value was given as 0. A larger DAOH reflects a better outcome. The different versions of the DAOH metric are denoted DAOH30, DAOH90, DAOH180 and DAOH365 and correspond to the 30-day, 90-day, 180-day and 365-day follow-up intervals, respectively.

The separate components of the DAOH were also derived, namely postoperative mortality, readmission and days out of hospital over the defined period. A continuous inpatient spell statistic for the index admission (CIPS) was calculated as the time from admission to final discharge to the community or non-acute care, and included the number of days within the neurosurgical unit as well as any days in another NHS hospital if the person was transferred as one continuous care episode. The postoperative death and readmission measures were binary variables, with a value of 1 if a person died or had one or more readmissions to hospital after the index admission within the specified DAOH follow-up interval (0 otherwise). The number of days in hospital for readmissions covered all hospital overnight and same day admissions over the defined period.

### Explanatory variables

Explanatory variables were derived from data related to each patient’s index admission and included patient age on admission, sex, a measure of frailty and comorbidity burden, and area deprivation score.

Comorbidity burden was measured using the Elixhauser Comorbidity Score (van Walraven modification), with weighted scores divided into six groups (−11 to −1, 0, 1 to 4, 5 to 8, 9 to 12, 13+).^18^ The comorbidity score was derived using a 1-year ‘look back’ period in order to differentiate between those diagnoses that were chronic conditions and should be classified as comorbidities compared with acute illnesses. Frailty was measured using the Secondary Care Administrative Records Frailty (SCARF) index.^19^ This index is based upon the model of frailty that characterises it as an ‘accumulation of deficits’. ICD-10 codes are used to define 32 deficits which cover functional impairment, geriatric syndromes, problems with nutrition, cognition and mood as well as medical comorbidities. It then categorises patients into ‘fit’, ‘mild frailty’, ‘moderate frailty’ and ‘severe frailty’.

### Statistical analysis

Summary statistics were derived to describe the cohort of patients, with the median and IQRs reported for continuous data and number/percentage for categorical variables. Histograms of DAOH for the time points of 30/90/180/365 days were generated to illustrate the differences in the distribution of scores. The output for ‘0 days’ DAOH was split into deaths and those remaining an inpatient over the entire period.

To examine the construct validity of the DAOH, we investigated the degree to which the DAOH discriminated between patients with different ages, levels of comorbidity and degree of frailty. We expected to observe worse values on these variables associated with increased perioperative risk. A Kruskal-Wallis test was performed to test whether differences in the median DAOH values across different variable categories were statistically significant.

As recommended in previous studies,^2 3 20^ we decided upon the preferred follow-up period for the DAOH outcome for CSDH patients based on pragmatic considerations and then also the observed behaviour of the DAOH at the different time points. We selected 90 days as the primary DAOH metric because it balanced the timely outcome capture with inclusion of the index admission CIPS for the vast majority of patients, while reducing the influence of unrelated readmissions that can accrue over longer horizons.

For the investigation of the DAOH90 metric when reported at an organisational level, we defined three different (adjusted) outcome indicators for all 24 NHS neurosurgery units. The statistics were:

1. Postoperative mortality rate at 90 days.
2. Average number of days in hospital for all patients at 90 days (including patients who died).
3. DAOH90 metric.

Unit-level outcomes were adjusted using a regression model that included patient age, sex, comorbidity status, frailty and whether a patient had a reoperation. Age was treated as a continuous variable and modelled with linear and quadratic terms to allow for non-linearity in its relationship with the outcome. A multivariable logistic regression was used for the postoperative mortality rate (binary outcome) and a multivariable linear regression for the average number of days in hospital and DAOH90 metric.

Indirect standardisation was used to produce risk adjusted outcomes for the NHS neurosurgery units, with the regression models being used to estimate expected number of events (postoperative deaths, readmissions) at each organisation given their casemix. Mixed-effects models were initially explored, but between-unit variance was minimal (intraclass correlation <2%), so final models were simplified to single-level regressions to aid interpretability. Variation in the risk-adjusted outcomes across the units was examined using a funnel plot. Performance that fell outside the expected range was indicated by 95% and 99.8% control limits plotted around the overall mean rate. Units with values outside the 99.8% control limits were considered statistical outliers as no unit would be expected to fall beyond the control limit due to chance alone.

### Patient and public involvement

This study did not involve patient or public involvement in its design, conduct, reporting or dissemination.

## Results

The study identified 16 450 patients who underwent burr-hole drainage of a CSDH between 1 April 2013 and 31 March 2020. The cohort consisted of 70.5% men and 29.5% women, and 69.2% were aged 70 years or over. Overall, 51.1% had an Elixhauser Comorbidity Scale of 1 or more, and 64.3% were rated as being moderately or severely frail on the SCARF index.

Table 1 summarises the findings for DAOH and its constituent metrics. For the DAOH derived from the first 30 days of postoperative follow-up, the median DAOH for patients with CSDH was 16 (IQR: 0 to 24). The distribution of length of stay (LOS) over the first 30 days after surgery had a median of 13 days (IQR: 6 to 29). The median LOS for the index admission in the neurosurgery unit was 8 days (IQR: 5 to 15 days), but including transfers to other hospitals, the CIPS extended beyond 30 days for a sizeable proportion of patients. The median CIPS was 14 days and the IQR extended from 7 to 33 days (table 2). The 30-day postoperative mortality rate was 5%.

**Table 1:** Summary of DAOH measure and constituent parts over different follow-up periods

Follow-up period (days)	DAOH, median (IQR) days	In-hospital mortality during index CIP, n (%)	Overall mortality, n (%)	Readmission, n (%)	Total LOS over period, median (IQR) days
CSDH (n=16 450)					
30	16 (0–24)	521 (3.2%)	880 (5%)	4020 (24%)	13 (6–29)
90	74 (42–84)	549 (3.3%)	1567 (10%)	4976 (30%)	14 (6–34)
180	163 (123–173)	553 (3.4%)	2049 (12%)	5729 (35%)	15 (6–38)
365	345 (288–358)	553 (3.4%)	2750 (17%)	6581 (40%)	16 (7–41)

CIP, continuous inpatient spell; CSDH, chronic subdural haematoma; DAOH, days alive and out of hospital; LOS, length of stay.

**Table 2:** Summary of DAOH values measured at 30, 90, 180 and 365 days for selected patient risk factors

Characteristics	No. of individuals (%)	DAOH30 Median (IQR)	DAOH90 Median (IQR)	DAOH180 Median (IQR)	DAOH365 Median (IQR)
Total (all)	16 450	16 (0–24)	74 (42–84)	163 (123–173)	345 (288–358)
Age (years)					
16–59	2530	20 (0–26)	79 (40–85)	168 (122–175)	351 (293–360)
60–69	2542	23 (8–26)	82 (63–86)	171 (150–175)	355 (327–360)
70–79	4878	19 (2–25)	78 (55–85)	167 (139–174)	350 (312–359)
80–89	5479	10 (0–21)	66 (32–80)	153 (106–169)	334 (243–353)
90+	1021	0 (0–15)	55 (9–73)	139 (0–162)	316 (0–344)
Sex					
Female	4857	13 (0–23)	75 (43–84)	164 (124–174)	346 (290–358)
Male	11 592	17 (0–25)	71 (41–83)	160 (121–172)	341 (283–356)
Elixhauser Comorbidity Score					
<0	1038	20 (3–25)	78 (58–85)	167 (144–175)	350.5 (319–359)
0	7006	20 (1–25)	79 (54–85)	169 (141–175)	353 (318–360)
1–4	2017	18 (0–24)	76 (54–84)	165 (138–174)	347 (311–358)
5–8	3261	12 (0–22)	70 (37–82)	158 (116–171)	339 (275–355)
9–12	1538	7 (0–20)	62 (16–78)	148 (69–166)	325 (0–349)
13+	1590	3 (0–17)	54 (0–75)	134 (0–162)	292 (0–342)
SCARF index					
Fit	2009	25 (19–26)	84 (78–86)	174 (167–176)	359 (350–361)
Mild frailty	3864	22 (10–26)	82 (66–85)	171 (154–175)	355 (334–360)
Moderate frailty	5280	15 (0–24)	74 (45–83)	162 (129–173)	345 (298–357)
Severe frailty	5297	2 (0–17)	56 (7–75)	140 (49–163)	314 (0–345)

The variation in the median scores across the categories was statistically significant (p<0.01) for all variables on the four DAOH measures (Kruskal-Wallis test).

DAOH, days alive and out of hospital; SCARF, Secondary Care Administrative Records Frailty.

**Figure 1:**
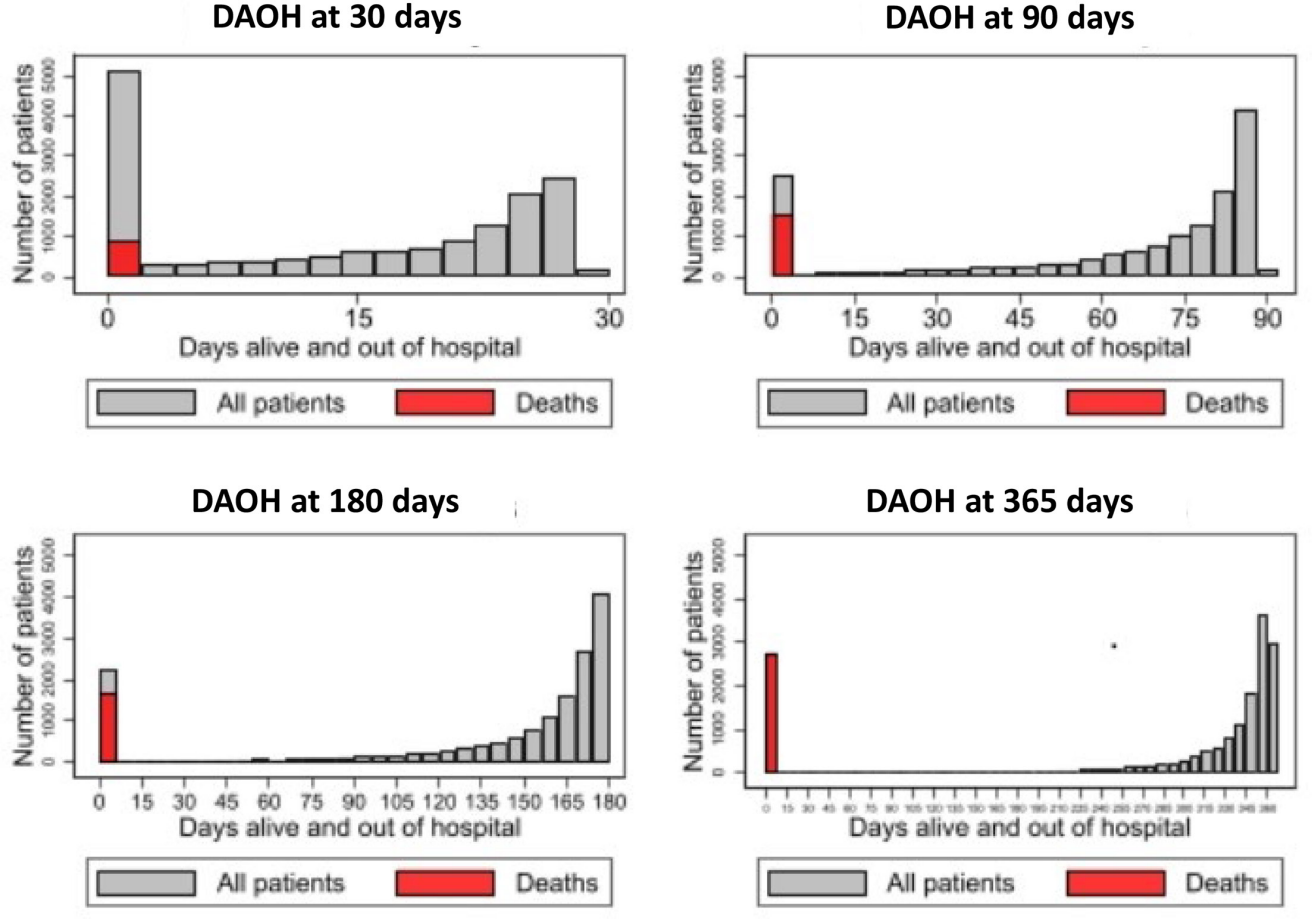
Histograms of the distribution of DAOH measured at 30, 90, 180 and 365 days for patients with CSDH. CSDH, chronic subdural haematoma; DAOH, days alive and out of hospital.

Events in the period after discharge from the index admission were relatively common. The mortality rate increased to 17% at 1 year from surgery and 40% of patients had been readmitted to hospital at least once (table 1). The additional number of days spent in hospital during these readmissions was not large, with a median LOS of 4 days (IQR: 1–11 days) for time spent at the neurosurgery hospital, and a median continuous inpatient stay of 8 day (IQR: 3–22 days). The distributions of DAOH at the four different times for patients with CSDH are shown in figure 1. All DAOH measures had a left-skewed distribution, with a secondary peak at 0 days. For the DAOH30 measure, the peak at 0 days was a mix of people who had died and who were still in hospital. For the DAOH measures based on longer periods, the peak at 0 days consisted predominantly of people who had died.

### Construct validity of days alive and out of hospital at different time periods

We examined the construct validity of the DAOH measure for patients with CSDH by exploring whether it behaved as expected across different perioperative risk factors. Table 2 shows the variation in DAOH values derived at the four different follow-up periods.

All DAOH measures had lower median values as the age of patients increased above 70 years, with the difference across the median values becoming proportionally larger for the DAOH with longer follow-up intervals. For the DAOH30 measure, the median dropped from 23 among patients aged 60–69 years to 0 for patients aged 90+ years. The median value for patients aged under 60 years tended to be slightly lower than for the 60–69 years’ group. The pattern across men and women was not consistent for all DAOH measures. Male patients had a median DAOH30 of 17 while the median was 13 for female patients. For the other DAOH measures, the median was higher among women than among men.

For both the comorbidity and frailty variables, the different DAOH measures exhibited a decreasing median value for patients with higher index scores (with the exception of the group with negative Elixhauser comorbidity values). Mortality after 30 days also rose with greater comorbidity burden, from 4% among those with an Elixhauser score of 0 or less to 12% for those with a score of at least 13.

Given these results, the 90-day follow-up period was selected as the preferred metric. It had the advantage of being available without a long delay from the time of the procedure, while also catching the end of care associated with the index admission in all but a few cases. There were strong associations (in the expected direction) with age, comorbidity (Elixhauser) and frailty (SCARF).

### Association between the outcomes (days alive and out of hospital at 90 days, mortality and days in hospital) and patient characteristics

The relationships between the three outcome indicators (DAOH90, 90-day postoperative mortality and days in hospital at 90 days) and the various patient variables were similar across the three regression models (onlinesupplemental tables 1^3^). A higher risk of postoperative mortality was associated with the increasing age, and a greater burden of comorbidity and frailty. Similar relationships between worse outcome values and increasing age and greater comorbidity and frailty burdens were observed for the days in hospital indicator and DAOH90. Male sex and the occurrence of a reoperation were associated with statistically significant worse outcomes in terms of days in hospital and DAOH90 but not 90-day postoperative mortality.

### Variation across neurosurgery units in days alive and out of hospital, mortality and days in hospital indicators

**Figure 2:**
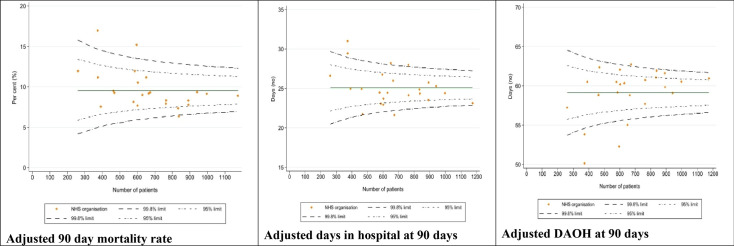
Funnel plot of variation in adjusted indicator values across neurosurgical units treating patients with CSDH. CSDH, chronic subdural haematoma; DAOH, days alive and out of hospital; NHS, National Health Service.

This section summarises the exploration of variation across NHS neurosurgery units on the three metrics. The funnel plots of risk-adjusted unit indicator values are shown in figure 2. For 90-day postoperative mortality, two units lay above the 99.8% upper control limit. Four units lay above the 99.8% upper control limit for risk-adjusted days in hospital within 90 days, and four units lay below the 99.8% lower control limit for DAOH90. The two mortality outliers were also among the units with the lowest DAOH90. Of the remaining two units with low DAOH90, both were above the 99.8% upper control limit for days in hospital. Only one unit lay outside the 99.8% control limits for all three metrics.

We examined the rank correlation of the unit values to examine the strength of association between the DAOH and its constituent outcomes. The ranking of units by DAOH90 was most strongly correlated with the days in hospital at 90 days (Spearman’s ρ=0.843) and was moderately correlated with postoperative mortality (ρ=0.647). Days in hospital and postoperative mortality showed a weak and non-significant association (ρ=0.278). Online supplemental figure 1 presents a heat map of unit rankings across the three metrics and demonstrates that, despite these associations, the individual units may rank differently depending on the outcome measure used.

## Discussion

This study was undertaken to investigate the validity of the DAOH outcome metric for patients undergoing burr-hole drainage of a CSDH. The cohort of CSDH patients represented a generally elderly population who required an emergency cranial procedure and may stay in hospital for an extended period after surgery as well as experience further hospital admissions. Among the four different versions of the DAOH metric, the DAOH based on a 90-day follow-up had the benefit of being available without a long delay, while also catching the end of care associated with the index admission in all but a few cases. It showed the expected relationships with age, comorbidity (Elixhauser) and frailty (SCARF), suggesting it had reasonable construct validity using a known-groups type methodology. The DAOH90 decreased among older patients, and as patients became more comorbid or frail. We note that people aged 16–59 years had lower DAOH values for all follow-up periods than people aged 60–69 years, which might be because this group of patients has more complex pathologies given that it is not common for people in this age group to suffer this condition. For example, younger patients suffering from polytrauma may wait in hospital for an acute subdural to chronicify.

When calculated for each neurosurgery unit, the risk-adjusted metrics of postoperative mortality, mean days in hospital and mean DAOH at 90 days showed similar patterns of variation, with most units having values within the expected range of the national rates. The funnel plots identified a number of positive and negative statistical outliers (outside the 99.8% control limit) and these differed slightly between the three metrics. All extreme statistical outliers for DAOH90 were also extreme outliers for at least one of mortality or days in hospital, and there were no outliers on the constituent metrics that were also not an outlier on the DAOH90. However, only one unit was an extreme outlier for all three metrics.

### Comparison with other studies and future work

The previous prospective, multicentre UK study sought to describe the current management and outcomes in this condition within neurosurgery units.^10^ It captured a series of separate outcomes including within-unit mortality, within-unit morbidity, symptomatic recurrence within 60 days and unfavourable functional status at neurosurgical unit discharge. However, neurosurgery is a tertiary specialty with complex patient pathways that may involve patients interacting with different institutions in addition to the hospital where an index procedure was performed,^21 22^ and a limitation of that study was its failure to capture these in an interpretable metric. The construct validity of the DAOH metric demonstrated in this study suggests that it is capable of capturing the complexity of these care trajectories and would complement these other typical metrics.

It has been suggested that the DAOH could be used for assessing hospital performance.^16^ They similarly found that mortality and DAOH data, for example, were not totally in agreement and concluded that DAOH has considerable potential for future use in measuring surgical outcomes. Organisational level DAOH statistics for English neurosurgery units are likely to meet the requirements of being valid and reliable, when derived using HES data linked to the ONS death register given the ability to identify individual patients across English NHS hospitals. However, a concern with using administrative datasets to derive performance indicators is whether casemix differences across units can be measured.^23^ A limitation of HES is that it does not contain important factors related to disease severity such as the Glasgow Coma Score (GCS) on admission for CSDH patients. One way to mitigate this limitation is to focus on within-unit comparison using a tool such as a cumulative sum (CUSUM) chart^24^ to track performance over time because casemix differences are less likely to introduce confounding. However, the DAOH metric is a highly skewed distribution, bounded and with a peak at 0 due to deaths, which poses difficulties in defining boundaries to indicate acceptable or unacceptable performance. Consequently, it is likely that monitoring performance over time will remain focused on LOS and postoperative mortality because control charts like CUSUMs can be derived more easily. Further methodological work is needed to demonstrate the benefits of DAOH for performance assessment.

### Strengths and limitations

The study has various strengths. It used a population-based cohort of CSDH patients who had surgery. The HES dataset covers the activity of all NHS neurosurgery units in England and therefore provides a comprehensive picture of practice. Linkage with the ONS death register also means that the derived DAOH metrics for each follow-up period are unlikely to have omitted many deaths.

Our study has several limitations. First, the cohort was defined using the combination of operative codes but this may not pick up some patients because of incomplete coding and limitations of the OPCS classification. The operative code for subdural haematoma does not differentiate between chronic subdural and acute subdural haematoma and the craniotomy code is also not subdivided. This means that patients undergoing a mini-craniotomy for a subdural haematoma may not have been captured by this study, a group that made up 9% (72/787) of the patients within the UK prospective, multicentre cohort study.^10^ Second, in assessing construct validity, we were unable to examine the relationship between the DAOH metric and measures of morbidity since this is not something that is well captured within HES data. Another limitation of using an administrative dataset is its lack of some clinically relevant determinants of outcome such as GCS on admission or imaging characteristics. This means that, after adjustment for known confounders, there may remain some residual confounding that could explain variation in the risk-adjusted outcomes derived for the neurosurgical units. Finally, for the purposes of external validation, the metric requires evaluation on patients with CSDH within a different healthcare system and we hope other international groups will undertake this.

## Conclusion

The DAOH metric is being advocated as potentially useful for evaluating surgical outcomes. For patients undergoing burr-hole drainage for CSDH, it was feasible to derive using national hospital data and the version based on 90-day follow-up appears capable of capturing variation between patients and its behaviour was as expected across patients with different perioperative risk factors. Future work should explore how to estimate a minimally important clinical difference for DAOH and evaluate its utility as an outcome measure in neurosurgical clinical trials. It also has potential for monitoring outcomes across neurosurgical units but further work is required to assess the performance of risk-adjusted organisation-level DAOH statistics produced from datasets containing baseline disease severity before considering its use for benchmarking practice.

## Supplementary material

10.1136/bmjopen-2025-114095 online supplemental file 1

10.1136/bmjopen-2025-114095 online supplemental table 1

10.1136/bmjopen-2025-114095 online supplemental table 2

10.1136/bmjopen-2025-114095 online supplemental table 3

10.1136/bmjopen-2025-114095 online supplemental figure 1

## Data Availability

No data are available.
