# Supplementary material for: Days alive and out of hospital after burr-hole drainage for chronic subdural haematoma: a national cohort study using Hospital Episode Statistics in England
Source: BMJ Open. 2026 Apr 13;16(4):e114095. doi: 10.1136/bmjopen-2025-114095 (PMC13084945; doi:10.1136/bmjopen-2025-114095)
Supplement: online supplemental table 1 [file bmjopen-16-4-s002.docx]

**Supplementary Table 1: Coefficients from a logistic regression for the association of patient characteristics with 90-day postoperative mortality**

| Variable | Coefficient | Std. Error | z-value | p-value | 95% Confidence Interval | |
| --- | --- | --- | --- | --- | --- | --- |
| **Start Age** | -0.098 | 0.012 | -8.27 | <0.001 | | -0.121 to -0.075 |
| **Age Squared** | 0.001 | 0.0001 | 9.16 | <0.001 | | 0.0006 to 0.001 |
| **Male (vs. Female)** | 0.032 | 0.059 | 0.55 | 0.584 | | -0.083 to 0.147 |
| **Elixhauser Category** |  |  |  |  | |  |
| -10 - -1 | -0.197 | 0.143 | -1.38 | 0.167 | | -0.478 to 0.083 |
| 1–4 | -0.003 | 0.101 | -0.03 | 0.975 | | -0.201 to 0.195 |
| 5–8 | 0.199 | 0.081 | 2.45 | 0.014 | | 0.040 to 0.358 |
| 9–12 | 0.536 | 0.094 | 5.68 | <0.001 | | 0.351 to 0.720 |
| 13+ | 0.901 | 0.089 | 10.07 | <0.001 | | 0.725 to 1.076 |
| **SCARF Index** |  |  |  |  | |  |
| Mild Frailty | 0.353 | 0.144 | 2.37 | 0.014 | | 0.070 to 0.636 |
| Moderate Frailty | 0.702 | 0.139 | 5.04 | <0.001 | | 0.429 to 0.975 |
| Severe Frailty | 1.182 | 0.142 | 8.31 | <0.001 | | 0.903 to 1.460 |
| **Reoperation** | 0.140 | 0.086 | 1.63 | 0.102 | | -0.028 to 0.309 |
| **Constant** | -0.712 | 0.387 | -1.84 | 0.066 | | -1.471 to 0.048 |
