# Supplementary material for: Days alive and out of hospital after burr-hole drainage for chronic subdural haematoma: a national cohort study using Hospital Episode Statistics in England
Source: BMJ Open. 2026 Apr 13;16(4):e114095. doi: 10.1136/bmjopen-2025-114095 (PMC13084945; doi:10.1136/bmjopen-2025-114095)
Supplement: online supplemental table 2 [file bmjopen-16-4-s003.docx]

**Supplementary Table 2: Coefficients from a linear regression for the association of patient characteristics and days in hospital at 90-days**

| Variable | Coefficient | Std. Error | z-value | p-value | 95% Confidence Interval | |
| --- | --- | --- | --- | --- | --- | --- |
| **Start Age** | -1.434 | 0.084 | -17.14 | <0.001 | | -1.60 to -1.270 |
| **Age Squared** | 0.011 | 0.001 | 16.71 | <0.001 | | 0.009 to 0.012 |
| **Male (vs. Female)** | 1.383 | 0.405 | 3.42 | 0.001 | | 0.590 to 2.176 |
| **Elixhauser Category** |  |  |  |  | |  |
| -10 - -1 | -5.282 | 0.807 | -6.55 | <0.001 | | -6.864 to -3.701 |
| 1–4 | -3.846 | 0.618 | -6.23 | <0.001 | | -5.057 to -2.636 |
| 5–8 | -1.556 | 0.544 | -2.86 | 0.004 | | -2.621 to -0.491 |
| 9–12 | -0.627 | 0.718 | -0.87 | 0.383 | | -2.035 to 0.781 |
| 13+ | 0.230 | 0.728 | 0.32 | 0.752 | | -1.196 to 1.657 |
| **SCARF Index** |  |  |  |  | |  |
| Mild Frailty | 6.755 | 0.669 | 10.10 | <0.001 | | 5.445 to 8.066 |
| Moderate Frailty | 14.137 | 0.673 | 20.99 | <0.001 | | 12.817 to 15.457 |
| Severe Frailty | 23.146 | 0.734 | 31.54 | <0.001 | | 21.707 to 24.584 |
| **Reoperation** | 15.674 | 0.616 | 25.46 | <0.001 | | 14.467 to 16.881 |
| **Constant** | 55.835 | 2.692 | 20.74 | <0.001 | | 50.558 to 61.111 |
