# Supplementary material for: Days alive and out of hospital after burr-hole drainage for chronic subdural haematoma: a national cohort study using Hospital Episode Statistics in England
Source: BMJ Open. 2026 Apr 13;16(4):e114095. doi: 10.1136/bmjopen-2025-114095 (PMC13084945; doi:10.1136/bmjopen-2025-114095)
Supplement: online supplemental table 3 [file bmjopen-16-4-s004.docx]

**Supplementary Table 3: Coefficients from a linear regression for the association of patient characteristics and DAOH at 90-days**

| Variable | Coefficient | Std. Error | z-value | p-value | 95% Confidence Interval |
| --- | --- | --- | --- | --- | --- |
| **Start Age** | 1.891 | 0.0993 | 19.04 | <0.001 | 1.697 to 2.086 |
| **Age Squared** | -0.014 | 0.001 | -19.03 | <0.001 | -0.016 to -0.013 |
| **Male (vs. Female)** | -1.725 | 0.480 | -3.59 | <0.001 | -2.667 to -0.784 |
| **Elixhauser Category** |  |  |  |  |  |
| -10 - -1 | 5.581 | 0.958 | 5.82 | <0.001 | 3.703 to 7.459 |
| 1–4 | 3.736 | 0.733 | 5.10 | <0.001 | 2.299 to 5.174 |
| 5–8 | 0.675 | 0.645 | 1.05 | 0.296 | -0.590 to 1.940 |
| 9–12 | -2.713 | 0.853 | -3.18 | 0.001 | -4.385 to -1.040 |
| 13+ | -7.150 | 0.864 | -8.27 | <0.001 | -8.844 to -5.456 |
| **SCARF Index** |  |  |  |  |  |
| Mild Frailty | -7.469 | 0.794 | -9.41 | <0.001 | -9.026 to -5.913 |
| Moderate Frailty | -15.610 | 0.800 | -19.52 | <0.001 | -17.178 to -14.042 |
| Severe Frailty | -27.323 | 0.871 | -31.35 | <0.001 | -29.031 to -25.615 |
| **Reoperation** | -15.176 | 0.731 | -20.76 | <0.001 | -16.610 to -13.743 |
| **Constant** | 19.043 | 3.197 | 5.96 | <0.001 | 12.777 to 25.308 |
