# Supplementary material for: Days alive and out of hospital after burr-hole drainage for chronic subdural haematoma: a national cohort study using Hospital Episode Statistics in England
Source: BMJ Open. 2026 Apr 13;16(4):e114095. doi: 10.1136/bmjopen-2025-114095 (PMC13084945; doi:10.1136/bmjopen-2025-114095)

**Figure 3: Heatmap comparing the rankings of each Neurosurgical unit on the metrics of 90 day postoperative mortality, days in hospital at 90 days, and DAOH at 90 days for patients with CDSH/ Ranks coloured from best (green) to worst (red)**


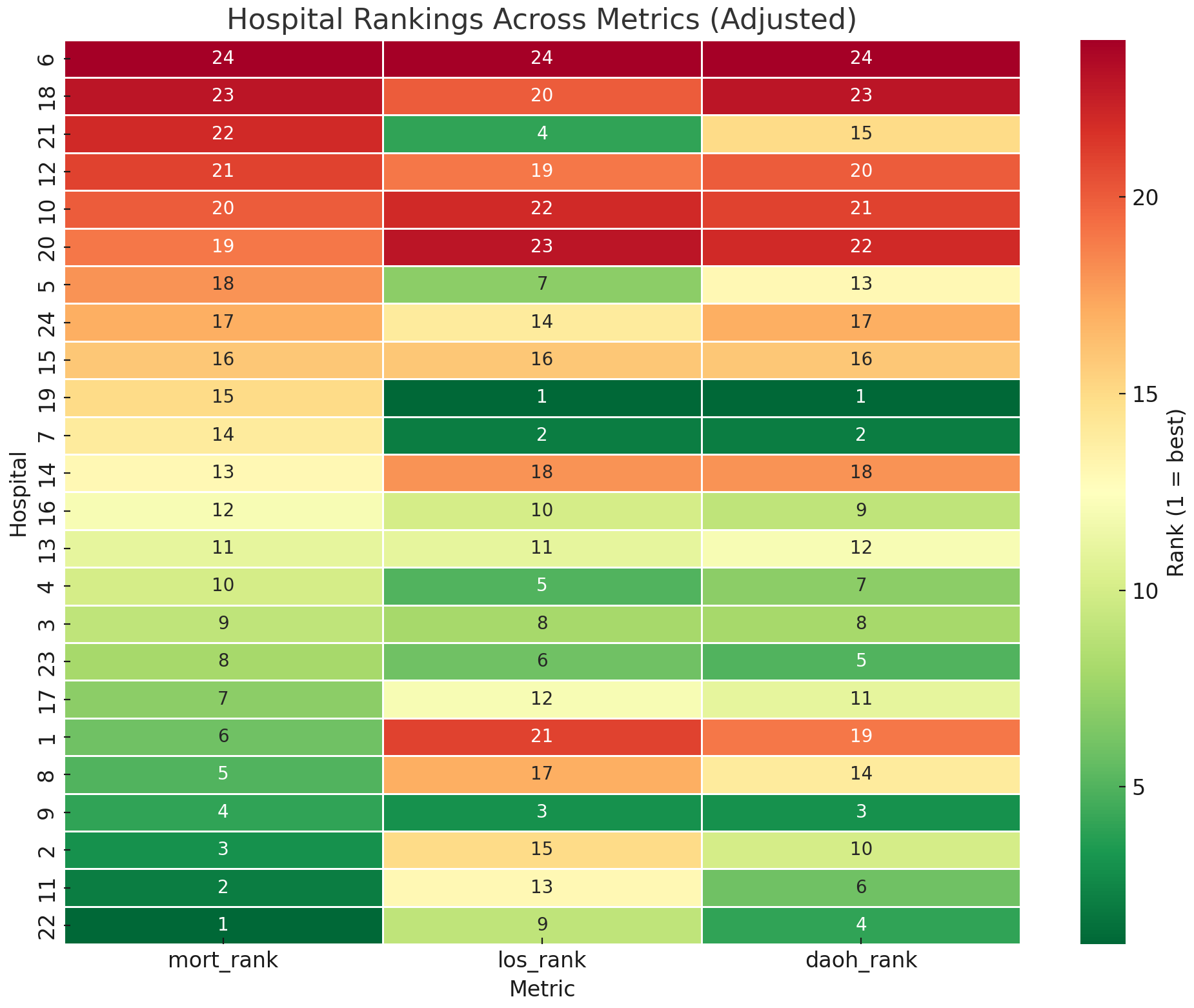

Supplement: online supplemental figure 1 [file bmjopen-16-4-s005.docx]
