# Supplementary material for: Days alive and out of hospital after burr-hole drainage for chronic subdural haematoma: a national cohort study using Hospital Episode Statistics in England
Source: BMJ Open. 2026 Apr 13;16(4):e114095. doi: 10.1136/bmjopen-2025-114095 (PMC13084945; doi:10.1136/bmjopen-2025-114095)
Supplement: Supplementary file 6 [file bmjopen-2025-114095.reviewer_comments.pdf]

PEER REVIEW HISTORY

BMJ Open publishes all reviews undertaken for accepted manuscripts. Reviewers are asked to complete a checklist review form and are provided with free text boxes to elaborate on their assessment. These free text comments are reproduced below.

ARTICLE DETAILS

Title (Provisional)

Days Alive and Out of Hospital after Burr-Hole Drainage for Chronic Subdural Haematoma: A National Cohort Study using Hospital Episode Statistics in England

Authors

Thompson, Daniel; Wahba, Adam; Davies, Benjamin; Williams, Adam; Hutchinson, Peter John; Stubbs, Daniel; Helmy, Adel; Cromwell, David A

VERSION 1 - REVIEW

**Reviewer**	**1**
**Name**	**Pirouzmand, Farhad**
**Affiliation**	**University of Toronto**
**Date**	**17-Dec-2025**
**COI**	**None**

Validation limited to CSDH: The study aim to validates the DAOH outcome measure only in the chronic subdural hematoma (CSDH) patient cohort. Including additional comparative cohorts (aneurysmal subarachnoid hemorrhage, ACDF, acoustic neuroma) is unnecessary and ultimately dilutes the clarity of the main CSDH findings.

Weak construct validity evidence: Construct validity for DAOH is demonstrated solely by an expected dose-response trend with known risk factors (increasing frailty, comorbid burden, and age). This is a relatively weak validation approach on its own, providing only minimal evidence that DAOH truly measures a meaningful patient-centered outcome.

DAOH adds little new insight: The 90-day DAOH metric correlates strongly with days in hospital (Spearman $\rho \approx 0.843$) and moderately with 90-day mortality ($\rho \approx 0.647$). Given these high correlations with existing metrics, DAOH90 appears to offer little novel information or performance insight beyond what length of stay and mortality already convey.

Arbitrary 90-day timeframe: The choice of a 90-day window for DAOH seems arbitrary and appears to have been decided post hoc. The manuscript provides no compelling clinical rationale for focusing on 90-day DAOH as opposed to other timeframes, which undermines

confidence that this interval is the most appropriate or meaningful for neurosurgical outcomes.

No severity adjustment: The DAOH metric is not adjusted for baseline neurological severity or disease acuity, which is a crucial determinant of outcomes in CSDH (and most cranial neurosurgery conditions). Therefore the DAOH comparisons may be confounded and fail to distinguish case-mix differences from true performance variation.

Unsubstantiated benchmarking claims: Claims that DAOH improves inter-hospital performance comparisons are not convincingly supported by the results. In practice, the neurosurgical units identified as outliers by DAOH90 are largely the same units flagged by traditional metrics like mortality or length of stay, suggesting that DAOH does not meaningfully enhance the detection of performance differences between hospitals.

Lack of patient-centered outcomes: The study omits any patient-reported outcome measures or functional outcome data, which is a major gap for a purported “patient-centered” metric. DAOH only captures healthcare utilization (time spent alive out of hospital), so without complementary PROMs or functional scores, it is unclear whether higher DAOH truly equates to better recovery or quality of life from the patient’s perspective.

Inconsistent rankings across conditions: Hospital performance rankings based on DAOH vary inconsistently across different neurosurgical conditions. A hospital that ranks highly on DAOH for one condition (e.g. CSDH) can rank poorly for another (e.g. acoustic neuroma), indicating that DAOH is highly context-dependent. This inconsistency would make DAOH unsuitable for creating any composite departmental “league table” because a single aggregated ranking would be misleading.

Overstated conclusions and omitted limitations: The manuscript tends to overstate its conclusions about DAOH’s usefulness while overlooking key limitations. Notably, it does not truly discuss the constraints of using administrative data, the absence of adjustment for disease severity, or the lack of external validation of DAOH in other settings. Consequently, any claims about DAOH’s generalizability or its value for performance benchmarking should be markedly tempered.

**Reviewer**	**2**
**Name**	**Klimek, Markus**
**Affiliation**	**Erasmus MC, University Medical Centre, Anesthesiology**
**Date**	**22-Dec-2025**
**COI**	**None**

This is a very interesting and relevant paper for several reasons, among which risk-stratification and prognosis of neurosurgical patients and benchmarking between neurosurgical departments are the most interesting ones.

It is clear written, well structured and provides all relevant information.

I really enjoyed reading it.

VERSION 1 - AUTHOR RESPONSE

Reviewer: 1
Dr. Farhad Pirouzmand, University of Toronto

Comments to the Author:
Validation limited to CSDH: The study aim to validates the DAOH outcome measure only in the chronic subdural hematoma (CSDH) patient cohort. Including additional comparative cohorts (aneurysmal subarachnoid hemorrhage, ACDF, acoustic neuroma) is unnecessary and ultimately dilutes the clarity of the main CSDH findings.

- **We thank the reviewer for this comment. The comparative cohorts (aneurysmal subarachnoid haemorrhage, anterior cervical discectomy and fusion, and acoustic neuroma) have been removed from the manuscript to improve clarity and maintain focus on validation of DAOH within the CSDH cohort.**

Weak construct validity evidence: Construct validity for DAOH is demonstrated solely by an expected dose-response trend with known risk factors (increasing frailty, comorbid burden, and age). This is a relatively weak validation approach on its own, providing only minimal evidence that DAOH truly measures a meaningful patient-centered outcome.

- **We thank the reviewer for highlighting this important point. We agree that the observed dose–response relationships with age, comorbidity and frailty represent only one aspect of construct validity (known-groups validity), and do not in themselves establish that DAOH captures patient-centred recovery. As administrative data do not contain functional outcome measures or patient-reported outcome measures (PROMs), convergent validity could not be assessed in this study. We have revised the manuscript to explicitly describe the validation approach as known-groups construct validity and have added text to highlight that linkage to registry and PROMs data would be required to assess convergent validity and strengthen interpretation.**

DAOH adds little new insight: The 90-day DAOH metric correlates strongly with days in hospital (Spearman $\rho \approx 0.843$) and moderately with 90-day mortality ($\rho \approx 0.647$). Given these high correlations with existing metrics, DAOH90 appears to offer little novel information or performance insight beyond what length of stay and mortality already convey.

- **We agree that DAOH is mathematically related to its constituent components and that correlation with length of stay and mortality is**

expected. We have revised the manuscript to avoid overstating novelty and to clarify that DAOH is not intended to replace mortality or length of stay, but to integrate survival, prolonged hospitalisation and readmission into a single, directionally consistent outcome reflecting time spent alive outside hospital. We also highlight that some associations (for example, sex and reoperation) differed between DAOH and postoperative mortality, supporting the interpretation that these measures are not directly interchangeable.

Arbitrary 90-day timeframe: The choice of a 90-day window for DAOH seems arbitrary and appears to have been decided post hoc. The manuscript provides no compelling clinical rationale for focusing on 90-day DAOH as opposed to other timeframes, which undermines confidence that this interval is the most appropriate or meaningful for neurosurgical outcomes.

- **We thank the reviewer for this comment. We have revised the Methods section to provide an explicit justification for the choice of the 90-day window. Specifically, we selected 90 days as the primary reporting interval because it balances timely outcome capture with inclusion of the full index continuous inpatient spell (CiP) for the vast majority of patients, while minimising the influence of unrelated readmissions that increasingly accrue over longer follow-up periods.**

No severity adjustment: The DAOH metric is not adjusted for baseline neurological severity or disease acuity, which is a crucial determinant of outcomes in CSDH (and most cranial neurosurgery conditions). Therefore the DAOH comparisons may be confounded and fail to distinguish case-mix differences from true performance variation.

- **We agree with the reviewer that baseline neurological severity and disease acuity are important determinants of outcome in CSDH. Such variables (for example, Glasgow Coma Scale, neurological deficit and imaging characteristics) are not captured within administrative datasets and therefore could not be included in our risk-adjustment models. We adjusted for age, comorbidity, frailty and reoperation to partially account for case-mix differences, but recognise that residual confounding is likely and have expanded the limitations section to explicitly acknowledge this. This represents an inherent trade-off of using large, linked administrative datasets, which provide complete population coverage and long-term follow-up but limited clinical granularity.**

Unsubstantiated benchmarking claims: Claims that DAOH improves inter-hospital performance comparisons are not convincingly supported by the results. In practice, the neurosurgical units identified as outliers by DAOH90 are largely the same units flagged by traditional metrics like mortality or length of stay, suggesting that DAOH does not meaningfully enhance the detection of performance differences between hospitals.

- **We thank the reviewer for this comment. We have revised the manuscript to temper claims regarding benchmarking and now present DAOH primarily as a tool for exploratory, risk-adjusted unit-level monitoring rather than as a definitive performance comparator. We explicitly note that overlap with traditional metrics such as mortality and length of stay is expected, and that residual confounding – including the absence of baseline neurological severity adjustment – limits causal interpretation of between-unit differences.**

Lack of patient-centered outcomes: The study omits any patient-reported outcome measures or functional outcome data, which is a major gap for a purported "patient-centered" metric. DAOH only captures healthcare utilization (time spent alive out of hospital), so without complementary PROMs or functional scores, it is unclear whether higher DAOH truly equates to better recovery or quality of life from the patient's perspective.

- **We agree with the reviewer that DAOH does not directly measure neurological recovery, functional outcome or quality of life, and that this represents an important limitation. We have strengthened the limitations section to make explicit that DAOH is a survival- and healthcare utilisation–based proxy rather than a direct measure of patient-centred recovery. We also note that DAOH has been endorsed by the international Standardised Endpoints in Perioperative Medicine (STEP) initiative as a patient-centred outcome reflecting time spent alive and outside hospital, which patients consistently value. Linkage to functional outcome measures and PROMs would be required to assess convergent validity and improve interpretability.**

Inconsistent rankings across conditions: Hospital performance rankings based on DAOH vary inconsistently across different neurosurgical conditions. A hospital that ranks highly on DAOH for one condition (e.g. CSDH) can rank poorly for another (e.g. acoustic neuroma), indicating that DAOH is highly context-dependent. This inconsistency would make DAOH unsuitable for creating any composite departmental "league table" because a single aggregated ranking would be misleading.

- **We agree with the reviewer that DAOH is context-dependent and that aggregating rankings across heterogeneous neurosurgical conditions could be misleading. In response to this concern, we have removed the additional pathologies from the manuscript and now focus exclusively on the CSDH cohort to maintain clinical coherence and clarity of interpretation.**

Overstated conclusions and omitted limitations: The manuscript tends to overstate its conclusions about DAOH's usefulness while overlooking key limitations. Notably, it does not truly discuss the constraints of using administrative data, the absence of adjustment for disease severity, or the lack of external validation of DAOH in other settings. Consequently, any claims about DAOH's generalizability or its value for performance benchmarking should be markedly tempered.

- **We thank the reviewer for this important comment. We have revised the manuscript to markedly temper the language used in the Discussion and Conclusions to avoid overstating the usefulness or generalisability of DAOH. We have expanded the limitations section to explicitly discuss the constraints of using administrative data, the absence of baseline neurological severity adjustment, and the lack of external validation in other healthcare settings. We have also reframed statements regarding benchmarking to emphasise the exploratory nature of unit-level analyses and the need for cautious interpretation. Taken together, these revisions clarify the scope of inference and ensure that conclusions are proportionate to the data available.**

Reviewer: 2
Dr. Markus Klimek, Erasmus MC, University Medical Centre

Comments to the Author:
This is a very interesting and relevant paper for several reasons, among which risk-stratification and prognosis of neurosurgical patients and benchmarking between neurosurgical departments are the most interesting ones.
It is clear written, well structured and provides all relevant information.
I really enjoyed reading it.

- **We thank the reviewer for their thoughtful and positive comments. We are pleased that the relevance, clarity and structure of the manuscript were well received. Revisions have been made in response to feedback from another reviewer, but the overall aims and focus of the study remain unchanged.**
